# AI-driven cognitive telerehabilitation for stroke: a randomized controlled trial

**DOI:** 10.3389/fneur.2025.1636017

**Published:** 2025-08-14

**Authors:** Suebeen Kim, Si-Woon Park, Taeksoo Jeong, Min-Soo Kang, Doo Young Kim

**Affiliations:** ^1^College of Medicine, Catholic Kwandong University, Gangneung, Republic of Korea; ^2^Department of Rehabilitation Medicine, International St. Mary’s Hospital, Incheon, Republic of Korea; ^3^Department of Physical and Rehabilitation Medicine, Seosong Hospital, Incheon, Republic of Korea

**Keywords:** artificial intelligence, cognitive dysfunction, mobile applications, stroke, telerehabilitation

## Abstract

**Background:**

Cognitive impairment is a common consequence of stroke, requiring effective rehabilitation strategies. Telerehabilitation has emerged as a promising alternative to in-person cognitive therapy, yet existing systems often lack mechanisms for real-time personalization and engagement monitoring. This study aimed to evaluate the clinical effectiveness of a self-guided AI-driven cognitive telerehabilitation compared to a therapist-supervised rehabilitation in subacute stroke patients.

**Methods:**

In this multicenter, parallel-group, randomized controlled non-inferiority trial, 63 participants with cognitive impairment within 6 months of stroke onset were randomized 1:1 to either a self-guided AI-driven telerehabilitation group or a therapist-supervised rehabilitation group. Both groups completed 24 sessions within 6 weeks using the same mobile platform. The primary outcomes were cognitive function measures, including the Korean Mini-Mental State Examination-2 (K-MMSE2), Trail Making Tests (A and B), and Digit Span Tests (forward and backward), with non-inferiority formally tested using the K-MMSE2. Secondary outcomes included functional independence, psychosocial measures and usability questionnaire.

**Results:**

Fifty-five participants completed the study. Both groups showed significant improvements across all primary cognitive measures, with no statistically significant differences between groups. Non-inferiority analysis confirmed that the self-guided AI-driven telerehabilitation was not inferior to the therapist-supervised rehabilitation based on K-MMSE2 changes. Usability assessment among users of the cognitive rehabilitation system indicated high overall satisfaction with no serious adverse events reported.

**Conclusion:**

This study demonstrates that a self-guided AI-driven telerehabilitation can deliver cognitive improvements comparable to a therapist-supervised rehabilitation in subacute stroke patients.

**Clinical trial registration:**

https://cris.nih.go.kr/cris/index/index.do, KCT0008969.

## 1 Introduction

Stroke is the second leading cause of death worldwide and ranks third in terms of disability-adjusted life years. While global stroke mortality rates have declined, the absolute number of stroke survivors continues to rise, leading to a growing population with post-stroke functional impairments who require long-term rehabilitation support (1, 2).

Among the diverse sequelae of stroke, cognitive impairment presents a major challenge due to its significant impact on daily functioning, independence, and overall quality of life (3). Impairments in memory, attention, and executive functioning hinder patients’ ability to carry out activities of daily living and increase the risk of developing post-stroke dementia. The resulting burden is not only clinical but also psychosocial and economic—families face increased caregiving responsibilities, emotional distress, and social isolation, while healthcare systems incur significant costs (4). Furthermore, as cognitive function plays a critical role in a patient’s ability to engage in rehabilitation programs, cognitive impairment may also limit the effectiveness of post-stroke recovery efforts (5, 6). Accordingly, cognitive rehabilitation has become an essential component of comprehensive stroke rehabilitation, alongside motor and functional rehabilitation (7).

Despite substantial demand for cognitive rehabilitation, conventional in-person modalities create substantial barriers to access, particularly for individuals with physical impairments or those residing in geographically underserved areas (8). Stroke survivors frequently encounter significant challenges in traveling to healthcare facilities, which may result in suboptimal adherence or premature discontinuation of therapy, even though clinical needs persist. The COVID-19 pandemic highlighted the need to develop alternative models for rehabilitation delivery, thereby catalyzing interest in telerehabilitation (9). Although telerehabilitation offers advantages such as reducing logistical burdens and enhancing continuity of care, its clinical utility remains limited. Challenges include monitoring patient engagement and dynamically adjusting task difficulty in the absence of direct therapist involvement (10, 11). Current guidelines, including those from the National Institute for Health and Care Excellence (NICE), support telerehabilitation only when it aligns with patient preferences and therapeutic goals, and they emphasize the need for ongoing evaluation of clinical efficacy and psychological well-being (12).

Computerized cognitive rehabilitation (CCR) was developed to address the limitations of traditional in-person cognitive therapy by providing structured, repeatable, and accessible interventions through digital platforms. CCR offers several advantages, including standardized training protocols, objective performance tracking, and the potential for self-guided therapy, which collectively supports cognitive recovery in a scalable manner (13). Several studies have found the effectiveness of CCR in many fields, such as healthy older adults, Parkinson’s disease, or multiple sclerosis (14–16). However, when applied in telerehabilitation contexts, conventional CCR systems still face notable challenges. These include difficulties in monitoring patient engagement remotely and the inability to adapt difficulty dynamically without therapist input, both of which can compromise the effectiveness of home-based telerehabilitation.

To overcome these limitations, advanced cognitive rehabilitation platforms that integrate cloud computing and artificial intelligence (AI) have recently emerged, including mobile and cloud-based AI-driven cognitive rehabilitation systems. These systems are specifically designed to enhance telerehabilitation by enabling real-time monitoring of patient participation through cloud infrastructure and by employing AI-driven algorithms that automatically adjust the complexity of training tasks based on individual performance data. Such innovations reduce the dependency on continuous therapist oversight while maintaining the quality and personalization of care. Collectively, these technologies facilitate the effective delivery of home-based, personalized cognitive telerehabilitation, thereby expanding access to rehabilitation services for patients who face barriers to traditional care.

Although AI-based telerehabilitation platforms are gaining attention, few have undergone thorough clinical evaluation. Most existing computerized cognitive rehabilitation programs follow a fixed sequence of tasks and require therapists to adjust difficulty levels or monitor patient adherence. In contrast, the system used in this study collects performance data in real time, including accuracy, response time, and task completion. It then uses this information to predict future performance and select appropriate training tasks, without the need for therapist involvement. This allows for cognitive training that is both adaptive and independently managed. Accordingly, this study aims to evaluate the effectiveness of self-guided AI-driven telerehabilitation in comparison to therapist-supervised rehabilitation in subacute stroke patients with cognitive impairment and offers evidence on the clinical feasibility through a multicenter randomized controlled trial.

## 2 Materials and methods

### 2.1 Materials

#### 2.1.1 Participants

Patients were eligible for enrollment if they were in the subacute phase of stroke, defined as within 6 months of onset, and exhibited cognitive impairment, as indicated by the Korean version of the Mini-Mental State Examination 2 (K-MMSE2) score of 26 or lower. Both ischemic and hemorrhagic stroke patients were included.

Exclusion criteria comprised patients with significant visual or auditory impairments that could interfere with the use of computerized devices, those with insufficient digital literacy (i.e., inability to operate smart devices effectively), individuals diagnosed with severe psychiatric disorders (e.g., schizophrenia, dissociative disorders, or major depressive disorder), and those taking antipsychotic medications, antidepressants, anti-alcohol medications, sedatives (e.g., benzodiazepines, tranquilizers), or opioids. Patients who did not provide IRB-approved written informed consent or refused to comply with the clinical study protocol were also excluded.

To ensure adequate participation, only patients who completed at least two-thirds (16 out of 24) of the scheduled intervention sessions were included in the final analysis. Those who participated in fewer than 16 sessions were considered dropouts and excluded from the study.

**Figure 1:**
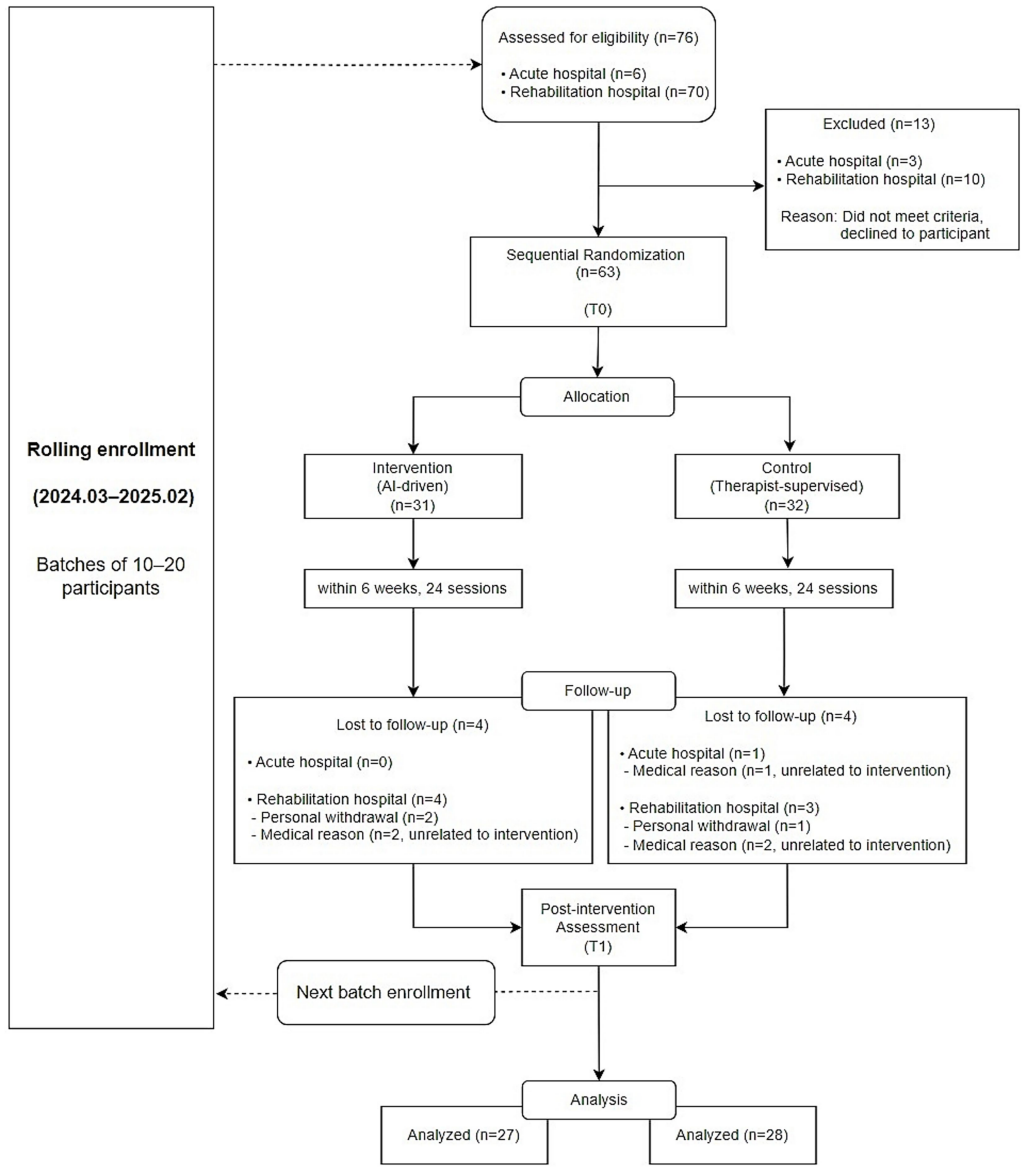
Overview of study design, rolling enrollment process, and CONSORT flow diagram.

Figure 1 shows the rolling enrollment process and sequential allocation of participants throughout the study period. Patients were recruited between March 2024 and February 2025. Recruitment was challenging because the study targeted subacute stroke patients whose eligibility was strictly limited by the time from onset, and the intervention required a relatively long treatment period, allowing only a small number of participants to undergo the program at the same time. A high dropout rate was also anticipated. Therefore, the initial recruitment target was set higher to account for potential attrition. As the study progressed, patients completed the intervention sequentially. Once the required sample size was reached in each group based on completed cases, recruitment was concluded.

A total of 55 participants (27 in the intervention group and 28 in the control group) completed the study and were included in the final analysis. The average age of all participants was 66.76 ± 11.54 years, and 41.8% (*n* = 23) were female. The median time from stroke onset to intervention was 54 days [30, 87], confirming that all participants were within the subacute phase. There were no statistically significant differences in baseline characteristics between the two groups. Detailed participant demographics are summarized in Table 1.

**Table 1:** General and baseline characteristics.

	Total (*N* = 55)	Intervention (*N* = 27)	Control (*N* = 28)	*p*-value
Sex, *n* (%)				0.665
Female	23	10 (37.0%)	13 (46.4%)	
Male	32	17 (63.0%)	15 (53.6%)	
Lesion, *n* (%)				0.069
Both	9	4 (14.8%)	5 (17.9%)	
Lt	33	20 (74.1%)	13 (46.4%)	
Rt	13	3 (11.1%)	10 (35.7%)	
Type, *n* (%)				0.194
Hemorrhagic	20	7 (25.9%)	13 (46.4%)	
Ischemic	35	20 (74.1%)	15 (53.6%)	
Since onset (days)	54 [30; 87]	51 [28; 83]	55 [31; 89]	0.643
Age (years)	66.76 ± 11.54	68.0 ± 12.5	65.6 ± 10.6	0.454
Education (years)	12 [6; 14]	12 [9; 15]	12 [6; 13]	0.306
CCI	1 [0; 2]	1 [0; 2]	1 [0; 2]	0.965
Smoke, *n* (%)				0.126
Current	11	6 (22.2%)	6 (17.9%)	
Ex-smoker	10	2 (7.4%)	8 (28.6%)	
Never	34	19 (70.4%)	15 (53.6%)	
Alcohol, *n* (%)				0.46
Heavy	3	1 (3.7%)	2 (7.1%)	
Never	24	10 (37.0%)	14 (50.0%)	
Social	28	16 (59.3%)	12 (42.9%)	
MMSE (T0)	22 [15; 25]	23 [17; 25]	19 [14; 23]	0.123
DSF (T0)	5 [4; 6]	6 [4; 7]	4 [4; 6]	0.208
DSB (T0)	2 [2; 3]	3 [2; 3]	2 [2; 3]	0.469
TMT-A (T0)	61.58 [33.69; 117.94]	48.58 [30.13; 91.65]	68.54 [45.40; 164.49]	0.082
TMT-B (T0)	134.63 [73.87; 300]	113.94 [70.65; 257.46]	152.44 [80.89; 300]	0.24
CES-D (T0)	16 [8; 32]	14 [7; 24]	23 [9; 34]	0.121
EQ-5D-5L (T0)	13.89 ± 5.11	13.11 ± 5.18	14.64 ± 5.01	0.27
SES (T0)	28 [25; 31]	30 [27; 33]	26 [22; 29]	0.033
MBI (T0)	47.67 ± 22.40	49.59 ± 23.12	45.8 ± 21.9	0.538

Values, mean ± SD or median [1st quartile; 3rd quartile]; CCI, Charlson Comorbidity Index; MMSE, Mini-Mental State Examination; DSF, Digit Span Forward; DSB, Digit Span Backward; TMT-A, Trail Making Test Part A; TMT-B, Trail Making Test Part B; CE-SD, Center for Epidemiologic Studies Depression Scale; EQ-5D-5L, EuroQol 5-Dimension 5-Level Questionnaire; SES, Socioeconomic Status; MBI, Modified Barthel Index.

#### 2.1.2 Intervention

Participants in the intervention group completed 24 sessions of self-guided AI-driven telerehabilitation using Zenicog^®^ (Mindhub Inc., Anyang, Republic of Korea) within a six-week period, with one session per day, each lasting approximately 30 min. The telerehabilitation program was delivered in a hospital setting, with participants performing the training sessions independently in their inpatient rooms without therapist supervision. Although the sessions were conducted within the hospital, the program utilized a non-face-to-face digital platform, fulfilling the criteria of cognitive telerehabilitation by enabling asynchronous, individualized training without real-time therapist involvement. The program employed an AI–powered recommendation engine that dynamically generated individualized training plans based on each participant’s real-time performance. The AI system comprised four key modules:

1. **Training record collection module**: This module gathered training session records, including accuracy rates and response times, to track the patient’s progress.
2. **Training question analysis module**: This module referenced a structured set of training tasks categorized by difficulty level and time limit, comparing them with the patient’s performance data to help identify appropriately challenging tasks.
3. **Training performance prediction module**: An AI model analyzed the patient’s past performance and predicted future training performance.
4. **Training recommendation module**: This module applied optimization algorithms to recommend appropriate training tasks, dynamically adjusting session content based on real-time performance.

The AI-based recommendation model employed in this study was not trained using big data or deep learning techniques. Rather, it recommends appropriate training tasks based on participants’ real-time performance data by selecting items from a training problem bank while considering factors such as task difficulty and time limit. Specifically, the model utilizes data including accuracy rate, response time, task difficulty, time limit, and number of attempts to assess each participant’s task-specific performance and predict future outcomes in real time. Based on this assessment, the system dynamically recommends subsequent training tasks. As this algorithm constitutes a rule-based system that generates recommendations from individual performance records, it does not require hyperparameter tuning or large-scale training datasets typically associated with deep learning models. Although external validation or generalizability testing of the model has not yet been conducted, qualitative evaluation based on user feedback and clinical observations has provided preliminary evidence supporting the clinical feasibility and practical utility of the algorithm. This approach enables real-time adjustment of training content according to participants’ evolving cognitive status without therapist involvement, thereby supporting continuous, individualized cognitive rehabilitation.

**Figure 2:**
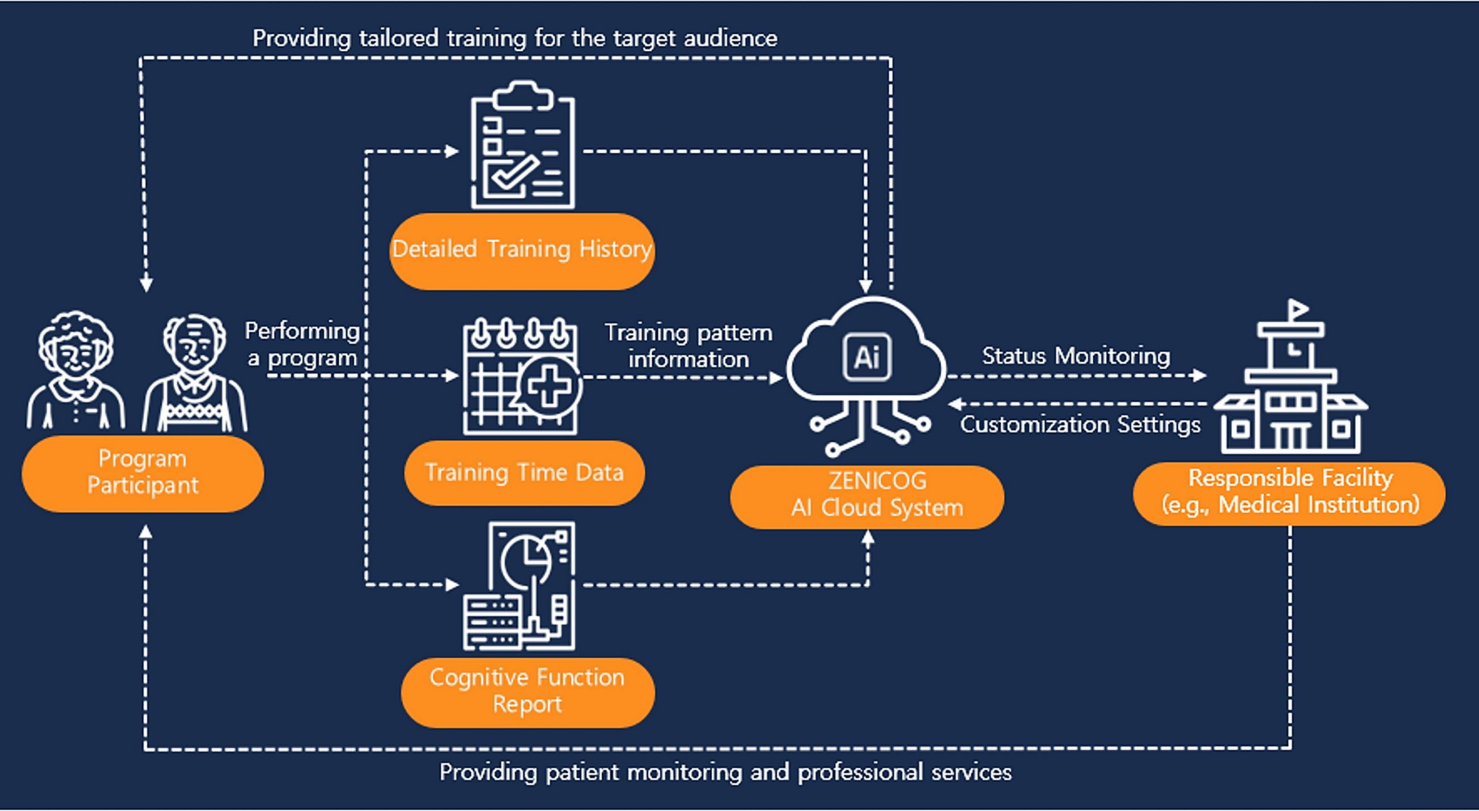
Schematic overview of the Zenicog® for AI-based cognitive rehabilitation.

Figure 2 shows how the cloud-based, AI-driven cognitive rehabilitation platform was used to monitor participant engagement and training patterns. This system enabled the research team to remotely track participants’ interactions with the platform, providing valuable data to inform future data-driven decision-making in cognitive rehabilitation.

Participants in the intervention group performed the training independently without therapist supervision, while maintaining conventional rehabilitation therapy at the same intensity as the control group.

In contrast, the control group received therapist-supervised cognitive rehabilitation, completing 24 sessions within 6 weeks using the same system. In this mode, therapists selected training tasks manually based on clinical judgment, and the AI-based recommendation engine was deactivated. All sessions were conducted in-person at the rehabilitation facility.

### 2.2 Methods

**Figure 3:**
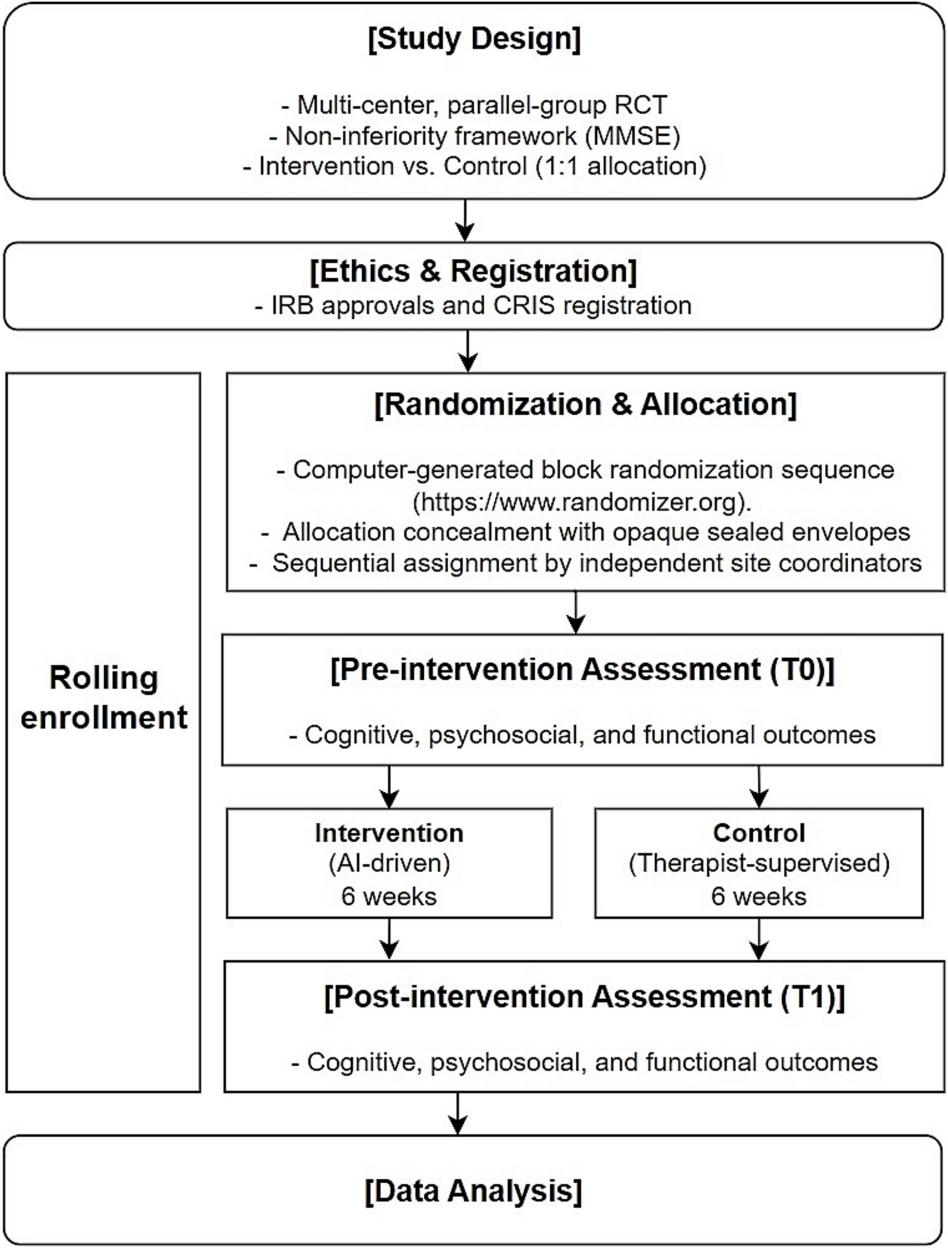
Study flow and methodological framework.

Figure 3 provides an overview of the entire study process, including design, ethical approval, randomization, intervention, outcome assessment, and analysis.

#### 2.2.1 Study design

This study was a multi-center, parallel group, randomized controlled trial designed to evaluate the effectiveness of a self-guided AI-driven telerehabilitation in comparison to therapist-supervised rehabilitation in subacute stroke patients with cognitive impairment. Participants were randomly assigned to either the intervention group (self-guided AI-driven telerehabilitation) or the control group (therapist-supervised rehabilitation) in a 1:1 allocation ratio.

#### 2.2.2 Randomization and allocation concealment

Participants were randomized using a computer-generated block randomization sequence (https://www.randomizer.org). Allocation concealment was ensured using sequentially numbered, opaque, sealed envelopes prepared by an independent researcher not involved in participant assignment or intervention. The envelopes were opened by designated research coordinators at each site upon participant enrollment.

To minimize bias, the researcher responsible for generating the randomization sequence was blinded to the allocation process and the research coordinators conducting the allocation were blinded to the randomization sequence. Due to the nature of the intervention, blinding of participants and therapists was not feasible. However, outcome assessors were blinded to group allocation and did not participate in intervention administration.

#### 2.2.3 Sample size calculation

This randomized controlled trial aimed to compare the effectiveness of self-guided AI-driven telerehabilitation with that of therapist-supervised cognitive rehabilitation. Among the prespecified primary cognitive outcomes, the Mini-Mental State Examination (MMSE) was selected as the reference measure for sample size estimation. For this outcome, a noninferiority hypothesis was applied to determine whether the self-guided intervention would not result in clinically unacceptable inferiority compared to the supervised approach. The calculation followed a standard formula for non-inferiority trials, referencing established statistical methodologies (17). The calculation was based on previously reported mean improvements in cognitive function and corresponding standard deviations. A mean MMSE score of 22 and a standard deviation of 5.19 were assumed, based on previously reported data from subacute stroke patients undergoing cognitive rehabilitation. The non-inferiority margin was set at 4, corresponding to the minimal clinically important difference (MCID) for the MMSE (18) With a one-sided alpha level of 0.025 and a power of 80% (beta = 0.20), the required sample size was determined to be 27 participants per group, totaling 54 participants.

#### 2.2.4 Outcome measures

This study evaluated the effectiveness of a self-guided AI-driven telerehabilitation program compared to therapist-supervised rehabilitation in subacute stroke patients. According to the pre-registered study protocol, outcome measures were categorized as primary and secondary outcomes. While non-inferiority was formally tested using the K-MMSE2, several other cognitive assessments were also designated as primary outcomes, enabling for a more comprehensive evaluation of domain-specific cognitive changes beyond global function.

##### 2.2.4.1 Primary outcomes: cognitive function

The primary outcomes focused on changes in cognitive function, assessed through the following measures:

K-MMSE2: This served as the primary endpoint for non-inferiority analysis. It assesses global cognitive function across seven domains—registration, orientation to time and place, recall, attention and calculation, language, and drawing. The total score ranges from 0 to 30, with higher scores indicating better cognitive performance.

Trail Making Test A and B (TMT-A & TMT-B): These tests assess visual attention, processing speed, and executive function. TMT-A involves connecting numbers 1 through 25 sequentially (e.g., 1-2-3-4), while TMT-B requires alternating between numbers and letters (e.g., 1-A-2-B). Completion times (in seconds) are recorded, with longer times indicating greater impairment. For participants unable to complete the tasks, maximum scores of 180 s (TMT-A) and 300 s (TMT-B) were assigned, consistent with standard scoring conventions (19).

Digit Span Test: This includes Digit Span Forward (DSF) and Digit Span Backward (DSB), which assess attention and working memory. Participants are asked to recall sequences of numbers in order (DSF) or in reverse (DSB), with lower scores indicating poorer performance.

##### 2.2.4.2 Secondary outcomes: psychosocial well-being, functional independence, and usability

The secondary outcomes encompassed psychosocial function, functional independence, and system usability:

5-level EQ-5D version (EQ-5D-5L): A standardized instrument assessing health-related quality of life across five domains: mobility, self-care, usual activities, pain/discomfort, and anxiety/depression. Higher index values reflect better perceived health status.

Self-Efficacy Scale (SES): Evaluated participants’ confidence in managing daily activities. Higher scores indicate greater self-efficacy.

Korean version of the Center for Epidemiological Studies–Depression Scale (K-CES-D): Assessed depressive symptoms, with higher scores indicating more severe depression.

Modified Barthel Index (MBI): Evaluated functional independence in activities of daily living (e.g., feeding, dressing, and mobility). Higher scores reflect greater independence.

System usability was assessed using a 25-item questionnaire developed based on Universal Design principles (20), encompassing domains such as equitable use and flexibility in use, simple and intuitive use, perceptible information, tolerance for error, low physical effort, size and space, overall product quality, overall satisfaction. Each item was rated on a 5-point Likert scale, with higher scores indicating better usability.

#### 2.2.5 Statistical analysis

All statistical analyses were conducted using SPSS (version 22.0; IBM Corp., NY, United States). The Kolmogorov–Smirnov test was used to assess the normality of continuous variables. For between-group comparisons, the independent *t*-test was used when the assumption of normality was met, and the Mann–Whitney U-test was applied otherwise. Categorical variables were compared using the chi-square test. Continuous variables are presented as mean ± standard deviation (SD) for normally distributed data, and as median with interquartile range [1st quartile; 3rd quartile] for non-normally distributed data.

For within-group comparisons of pre-and post-intervention values, the Wilcoxon signed rank test was used for non-normally distributed data, and the paired *t*-test was used when data followed a normal distribution. As a secondary analysis, time-to-event methods were used to address the presence of right-censored data in the TMT outcomes. Participants who failed to complete the TMT within the maximum allowed time (180 s for TMT-A and 300 s for TMT-B) were treated as censored cases. Cox proportional hazards regression was applied to compare the completion rates between groups, and Kaplan–Meier survival curves were generated to visualize task completion over time at both baseline and post-intervention time points.

Among the prespecified primary cognitive outcomes, only the change in MMSE score was analyzed under a non-inferiority framework. The non-inferiority margin was set at 4 points based on prior clinical considerations and literature. This analysis assumed normal distribution and was conducted using a one-sided independent *t*-test with a significance level of 0.025.

All other analyses, including comparisons of the remaining primary cognitive outcomes (such as TMT-A, TMT-B, DSF, DSB) and secondary outcomes, were performed to explore potential differences between groups. These analyses used two-sided tests with a significance level of 0.05 and were not based on a noninferiority or superiority design. All analyses of secondary outcomes were considered exploratory, and no correction for multiple comparisons was applied. Thus, these findings should be interpreted with caution.

#### 2.2.6 Ethical considerations

This multi-center study obtained Institutional Review Board (IRB) approvals from two institutions. The IRB approval for rehabilitation hospital (P01-202312-01-025) was acquired through the Public IRB system,^1^ while university-affiliated hospital has its own IRB (IS23OIME0062), from which separate approval was obtained. Additionally, the study was registered in the Clinical Research Information Service (CRIS^2^) prior to participant enrollment (Registration number: KCT0008969). Written informed consent was obtained from all participants before enrollment. The consent process was conducted in a private setting by a physician investigator, ensuring participants had sufficient time to review and understand the study procedures, risks, and benefits. In cases where participants had difficulty understanding, both the participant and their legal representative were provided with simultaneous explanations before obtaining consent.

## 3 Results

### 3.1 Study flow and retention

A total of 76 individuals were screened for eligibility across two participating institutions. Among them, 63 participants met the inclusion criteria and were randomized into the intervention or control group. The study targeted patients in the subacute phase after stroke, and eligibility was determined based on the time since onset. In addition, the intervention required a relatively long duration and only a limited number of participants could receive the intervention at the same time. Therefore, recruitment was carried out gradually, and the initial recruitment target was set higher to account for a potentially high dropout rate. As the study progressed, participants completed the intervention sequentially. Once the required number of participants had completed all sessions in each group, further recruitment was discontinued. During the study, 8 participants discontinued participation either due to personal withdrawal or medical reasons such as pneumonia or recurrent stroke. All medical events were unrelated to the intervention or device and were attributed to underlying health conditions commonly observed in the subacute stroke population. Similarly, all cases of personal withdrawal were voluntary and not associated with any adverse effects of the device. Therefore, no dropout was attributed to device-related side effects or complications. In the end, 55 participants (27 in the intervention group and 28 in the control group) completed the study and were included in the final analysis.

### 3.2 Results of cognitive, emotional, quality of life, and functional outcomes

Table 2 presents the within-group comparisons of cognitive and functional outcomes. In the TMT-A, a total of 11 participants who were unable to complete the task within the allotted time were assigned the maximum score of 180 s, in accordance with established scoring conventions (4 in the intervention group and 7 in the control group). For the TMT-B, 16 participants who did not complete the task were similarly assigned 300 s (6 in the intervention group and 10 in the control group). These values were used in the analysis based on the predefined scoring criteria.

**Table 2:** Within-group comparison of pre-and post-intervention changes.

	Total			Intervention			Control		
	Pre	Post	*p*-value	Pre	Post	*p*-value	Pre	Post	*p*-value
MMSE	22 [15; 25]	24 [19; 27]	<0.001*	23 [17; 25]	26 [21; 27]	<0.001*	19 [14; 23]	23 [18; 26]	<0.001*
DSF	5 [4; 6]	5 [4; 7]	0.016*	6 [4; 7]	6 [4; 7]	0.007*	4 [4; 6]	5 [4; 6]	0.226
DSB	2 [2; 3]	3 [2; 4]	0.009*	3 [2; 3]	3 [2; 4]	0.015*	2 [2; 3]	2 [2; 3]	0.183
TMT-A	61.58 [33.69; 117.94]	53.59 [31.94; 74.46]	0.001*	48.58 [30.13; 91.65]	47.39 [23.8; 72.43]	0.011*	68.54 [45.40; 164.49]	64.24 [39.42; 75.82]	0.026*
TMT-B	134.63 [73.87; 300]	102.22 [67.8; 168.31]	<0.001*	113.94 [70.65; 257.46]	81.25 [47.83; 141.19]	0.001*	152.44 [80.89; 300]	109.69 [79.56; 266.33]	0.014*
CES-D	16 [8; 32]	14 [5; 25]	0.037*	14 [7; 24]	10 [4; 20]	0.111	23.0 [9.0; 34.5]	18 [7; 33]	0.161
EQ-5D-5L	13.89 ± 5.11	13.33 ± 4.94	0.299	13.11 ± 5.18	13 ± 5.17	0.877	14.64 ± 5.01	13.64 ± 4.79	0.226
SES	28 [25; 31]	30 [24; 32]	0.477	30 [27; 33]	30 [26; 36]	1	26 [22; 29]	29 [22; 31]	0.353
MBI	47.67 ± 22.40	55.64 ± 20.79	<0.001*	49.59 ± 23.12	57.19 ± 21.33	<0.001*	45.8 ± 21.9	54.1 ± 20.5	<0.001*

Values, mean ± SD or median [1st quartile, 3rd quartile]; MMSE, Mini-Mental State Examination; DSF, Digit Span Forward; DSB, Digit Span Backward; TMT-A, Trail Making Test Part A; TMT-B, Trail Making Test Part B; CES-D, Center for Epidemiologic Studies Depression Scale; EQ-5D-5L, EuroQol 5-Dimension 5-Level Questionnaire; SES, Socioeconomic Status; MBI, Modified Barthel Index.

Within-group comparisons also showed statistically significant improvements in MMSE scores in both groups (*p* < 0.001). Improvements were also observed in other cognitive domains such as DSF, DSB, TMT-A, and TMT-B, with varying levels of statistical significance. Functional independence, as measured by the MBI, also improved significantly in both groups (*p* < 0.001).

Table 3 presents the between-group comparisons of post-intervention scores and change scores across cognitive, emotional, and functional outcomes. At post-intervention (T1), the median MMSE score was 26 [21; 27] in the intervention group and 23 [18; 26] in the control group (*p* = 0.177). No statistically significant differences were observed between groups in DSF (6 [4; 7] vs. 5 [4; 6], *p* = 0.213) or DSB (3 [2; 4] in both groups, *p* = 0.222). For TMT-A and TMT-B, median completion times were slightly shorter in the intervention group (TMT-A: 47.39 [23.8; 72.43] seconds vs. 64.24 [39.42; 75.82], *p* = 0.085; TMT-B: 81.25 [47.83; 141.19] seconds vs. 109.69 [79.56; 266.33], *p* = 0.142), although these differences were not statistically significant. No statistically significant between-group differences were observed in depressive symptoms (CES-D), quality of life (EQ-5D-5L), self-efficacy (SES), or functional independence (MBI).

**Table 3:** Comparison of treatment effectiveness between the two groups.

	Total (*N* = 55)	Intervention (*N* = 27)	Control (*N* = 28)	*p*-value
T1				
Completed session	24 [23; 24]	24 [23; 25]	24 [23; 24]	0.139
MMSE	24 [19; 27]	26 [21; 27]	23 [18; 26]	0.177
DSF	5 [4; 7]	6 [4; 7]	5 [4; 6]	0.213
DSB	3 [2; 4]	3 [2; 4]	3 [2; 4]	0.222
TM-A	53.59 [31.94; 74.46]	47.39 [23.8; 72.43]	64.24 [39.42; 75.82]	0.085
TMT-B	102.22 [67.8; 168.31]	81.25 [47.83; 141.19]	109.69 [79.56; 266.33]	0.142
CES-D	14 [5; 25]	10 [4; 20]	18 [7; 33]	0.058
EQ-5D-5L	13.33 ± 4.94	13 ± 5.17	13.64 ± 4.79	0.634
SES	30 [24; 32]	30 [26; 36]	29 [22; 31]	0.166
MBI	55.64 ± 20.79	57.19 ± 21.33	54.14 ± 20.53	0.592
T1-T0				
MMSE	2.76 ± 2.91	2.48 ± 2.81	3.04 ± 3.04	0.486
DSF	0 [0; 1]	0 [0; 1]	0 [0; 1]	0.614
DSB	0 [0; 1]	0 [0; 1]	0 [0; 1]	0.536
TMT-A	−5.29 [−19.27; 0]	−5 [−15.16; 0]	−5.73 [−21.09; 0]	0.846
TMT-B	−10.91 [−39.16; 0]	−17.09 [−39.16; −2.89]	−5.43 [−36.87; 0]	0.272
CES-D	−3.56 ± 12.37	−3.59 ± 13.83	−3.54 ± 11.04	0.987
SES	0 [−3; 3]	0 [−3; 2]	1 [−3; 4]	0.510
MBI	7 [0; 13]	3 [0; 13]	9 [0; 13]	0.416

Values, mean ± SD or median [1st quartile, 3rd quartile]; MMSE, Mini-Mental State Examination; DSF, Digit Span Forward; DSB, Digit Span Backward; TMT-A, Trail Making Test Part A; TMT-B, Trail Making Test Part B; CES-D, Center for Epidemiologic Studies Depression Scale; EQ-5D-5L, EuroQol 5-Dimension 5-Level Questionnaire; SES, Socioeconomic Status; MBI, Modified Barthel Index; CCI, Charlson Comorbidity Index.

In terms of change scores (T1–T0), both groups showed similar improvements. The mean MMSE change was 2.48 ± 2.81 in the intervention group and 3.04 ± 3.04 in the control group (*p* = 0.486). There were no significant group differences in changes in DSF, DSB, TMT-A, and TMT-B. Similarly, no significant between-groups were observed in changes in CES-D, SES, and MBI change scores.

### 3.3 Non-inferiority analysis

**Figure 4:**
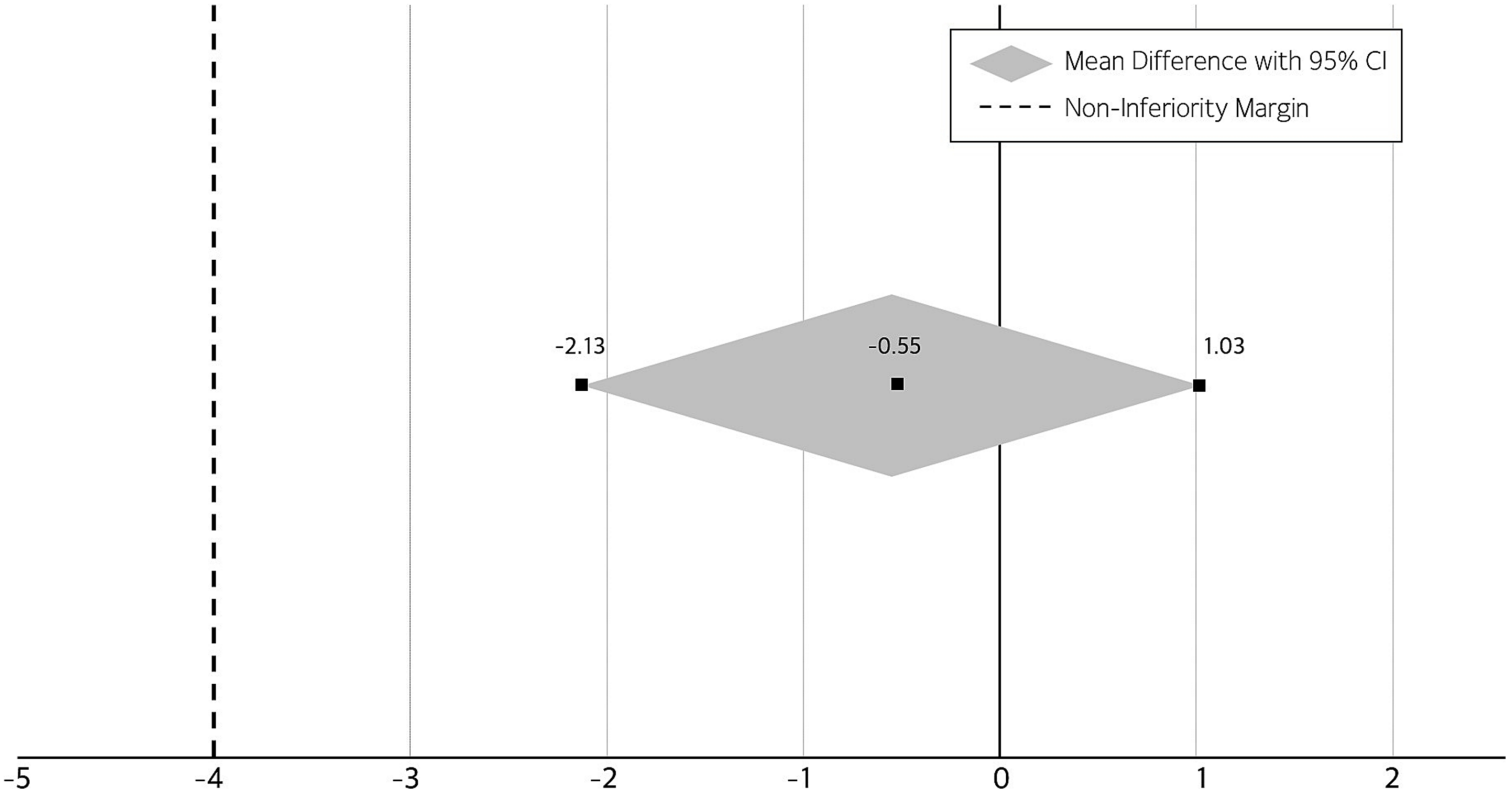
Non-inferiority analysis of the AI-based intervention compared to conventional therapy.

Figure 4 shows the results of the non-inferiority analysis comparing changes in MMSE scores between the two intervention groups. A non-inferiority analysis was conducted to compare the effectiveness of the self-guided AI-driven telerehabilitation with that of the therapist-supervised rehabilitation, based on changes in MMSE scores. Following the intervention, both groups demonstrated improvements in cognitive function. The mean change in MMSE score was 2.48 ± 2.81 in the intervention group and 3.04 ± 3.04 in the control group. The between-group mean difference (intervention minus control) was −0.55, with a 95% confidence interval of −2.13 to 1.03. As the entire confidence interval was above the pre-specified non-inferiority margin of 4, the AI-based intervention was determined to be non-inferior to the conventional therapist-supervised program.

### 3.4 Time-to-event analysis

**Figure 5:**
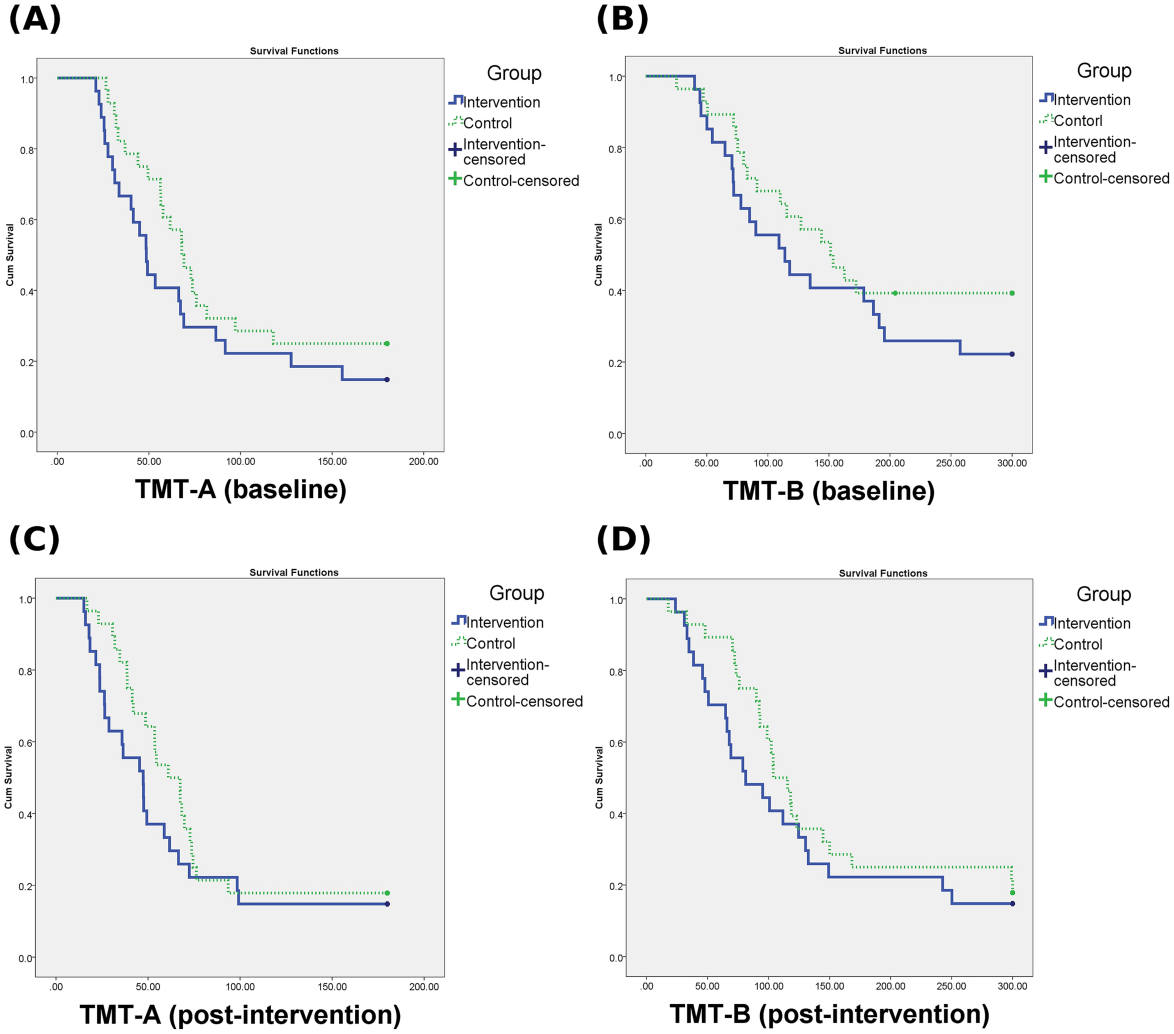
Kaplan–Meier survival curves for TMT-A and TMT-B. **(A)** TMT-A at baseline, **(B)** TMT-B at baseline, **(C)** TMT-A at post-intervention, **(D)** TMT-B at post-intervention. Curves represent the probability of task completion over time. Censored data indicate participants who did not complete the task within the maximum allotted time (180 s for TMT-A, 300 s for TMT-B).

Figure 5 shows the Kaplan–Meier survival curves illustrating task completion rates for TMT-A and TMT-B at baseline and post-intervention in both groups. To account for right-censoring in the TMT, additional time-to-event analyses were conducted using the Cox proportional hazards model at both baseline and post-intervention time points. At baseline, the AI group showed a non-significantly lower hazard of task completion compared to the therapist-supervised group in both TMT-A (HR = 0.660, 95% CI: 0.365–1.195, *p* = 0.17) and TMT-B (HR = 0.662, 95% CI: 0.349–1.255, *p* = 0.21). Similarly, at the post-intervention time point, the AI group also demonstrated non-significantly lower hazard rates for TMT-A (HR = 0.720, 95% CI: 0.403–1.286, *p* = 0.27) and TMT-B (HR = 0.724, 95% CI: 0.403–1.301, *p* = 0.28). These results suggest no statistically significant differences in TMT task completion rates between the two groups at either time point. The results are visualized using Kaplan–Meier survival curves.

### 3.5 System usability and user experience assessment

Table 4 presents the usability scores across eight domains for both the intervention and control groups. Usability was evaluated across eight domains using a structured questionnaire. Both the intervention and control groups showed similarly favorable responses across all domains, with no statistically significant differences. The median scores for overall product quality and overall satisfaction were 3.6 [3.2, 4.0] and 4.0 [3.3, 4.0], respectively. No major usability-related issues were identified, suggesting that the cognitive rehabilitation system was well accepted for use among the study population.

**Table 4:** Usability questionnaire responses.

	Total	Intervention (*N* = 27)	Control (*N* = 28)	*p*-value
Equitable use and flexibility in use	3.33 [2.67; 4]	3.33 [2.33; 4]	3.17 [2.67; 3.92]	0.529
Simple and intuitive use	3 [2.75; 4]	3.25 [2; 4]	3 [2.75; 3.69]	0.425
Perceptible information	4 [3; 4.5]	4 [3.5; 4.5]	3.5 [3; 4.5]	0.292
Tolerance for error	3.25 [3; 4]	3.5 [3; 4]	3.25 [3; 3.5]	0.213
Low physical effort	4 [3; 4]	4 [3; 4]	3.75 [3; 4]	0.796
Size and space for use	3.5 [2.5; 4]	3.5 [2; 4]	3.25 [2.63; 4.38]	0.688
Overall product quality	3.6 [3.2; 4]	3.8 [3.4; 4]	3.6 [3.2; 3.95]	0.218
Overall satisfaction	4 [3.3; 4]	4 [3.33; 4.33]	4 [3.33; 4]	0.231

Values, mean ± SD based on a 5-point Likert scale (1 = poor, 5 = excellent), with each item rated individually.

No significant adverse events that necessitated withdrawal from the study were observed in either group. Some participants, particularly older individuals, reported mild inconveniences such as poor visibility of on-screen text or difficulty using the touch interface; however, these were minor and did not affect overall adherence to the intervention.

## 4 Discussion

### 4.1 Summary of key findings

This study demonstrated that self-guided AI-driven telerehabilitation can lead to cognitive improvements comparable to those achieved with therapist-supervised rehabilitation. The self-guided AI-driven telerehabilitation group showed similar improvements in MMSE scores as the therapist-supervised rehabilitation group. Both groups achieving significant gains across cognitive and functional domains, including MMSE, DSF, DSB, TMT-A, TMT-B, and MBI. No statistically significant differences were observed between groups in performance. Participant satisfaction was high in both groups, and no serious adverse effects related to the intervention were identified throughout the study period.

### 4.2 Comparison with prior research

Telerehabilitation offers several potential advantages, such as reduced travel time, cost-efficiency, improved accessibility, and increased patient autonomy, which may be particularly helpful in underserved settings (21). A recent meta-analysis comparing telerehabilitation with face-to-face interventions reported comparable outcomes between the two approaches, with some benefits observed in domains such as working memory and executive function for the telerehabilitation group (11). A previous systematic review conducted by our team similarly found that cognitive telerehabilitation yielded outcomes comparable to conventional rehabilitation in global cognition, attention, and visuospatial functions, and showed some improvement in immediate global cognition when compared to usual care (22).

Although the efficacy of telerehabilitation was demonstrated, these studies did not utilize AI-driven systems. Despite growing interest in remote cognitive rehabilitation, only few studies have examined the effectiveness of AI-based telerehabilitation with minimal therapist involvement. To the best of our knowledge, this study is the first randomized controlled trial to investigate the clinical efficacy of an AI-integrated cognitive rehabilitation program. A previous exploratory study investigated patient experiences with an AI-supported rehabilitation application for stroke patients. Although patients’ responses were generally positive, the study was descriptive in nature and did not assess clinical efficacy. Rather, it provided preliminary insights into user attitudes and the feasibility of integrating AI into telerehabilitation, without offering evidence on therapeutic effectiveness (23).

### 4.3 Clinical feasibility and user experience

This study employed a comprehensive evaluation approach, including cognitive assessments (MMSE, TMT-A, Digit Span, TMT-B), functional independence (MBI), and psychosocial outcomes (CES-D, SES). The use of a structured usability questionnaire to identify user experience and areas for improvement further strengthens the study. Moreover, by adopting a RCT design—the highest level of evidence in evidence-based medicine—this study provides robust clinical evidence to support the efficacy of AI-based cognitive telerehabilitation (24). The system utilized in this study differs from conventional computerized cognitive rehabilitation platforms. Operating on a tablet PC, it incorporates a cloud-based monitoring system that enables clinicians to track patient progress remotely. The platform integrates four core AI modules—record tracking, training question analysis, performance prediction, and task recommendation—that personalize cognitive training based on real-time performance. This adaptive difficulty mechanism facilitates standardized and individualized telerehabilitation, enhancing accessibility and potential scalability in clinical practice. Notably, the results of this study suggest that self-guided AI-driven telerehabilitation delivered through such a system is not inferior in efficacy to traditional therapist-supervised rehabilitation. Comparable improvements were observed across key cognitive and functional outcomes, indicating that this technology-driven approach may serve as a viable alternative to in-person interventions, particularly in settings where conventional access is limited.

However, telerehabilitation may not be equally effective or appropriate for all patient populations. Individual factors such as age, digital literacy, and access to compatible technology can significantly influence both usability and treatment outcomes. Moreover, successful implementation requires adequate infrastructure, user training, and reliable technical support. In certain contexts, the initial setup cost for devices and digital platforms may also pose a barrier to broader adoption. Therefore, telerehabilitation should be viewed as a complementary tool rather than a universal replacement for conventional face-to-face cognitive therapy, especially in settings where in-person care is limited or unavailable.

Recent developments in virtual reality (VR)-based telerehabilitation have further expanded the scope of digital cognitive interventions. For example, one study demonstrated that immersive VR environments could significantly improve cognitive outcomes, such as Montreal Cognitive Assessment scores, in individuals with severe cognitive impairment, even with minimal therapist involvement (25). While promising, such approaches may also introduce new challenges, including increased costs, disorientation, or reduced tolerability among older adults or those unfamiliar with immersive technologies. In contrast, the system used in the present study demonstrated good usability and acceptability, with minimal concerns reported by participants. While a few older adults noted minor difficulties such as reduced visual clarity and challenges with touchscreen responsiveness, these issues were not severe enough to interfere with continued use or adherence. Notably, the usability scores did not differ significantly between the intervention and control groups, even though participants in the intervention group used the system independently without therapist supervision. This suggests that the platform can be effectively used by patients in a self-guided manner, without compromising user experience or accessibility. These findings indicate that the AI-driven telerehabilitation platform is well-tolerated in a subacute stroke population, supporting its potential for broader clinical application in cognitively impaired individuals with varying levels of technological familiarity.

### 4.4 Key contributions and practical relevance

These findings highlight the practical contributions of this study in addressing common challenges in cognitive telerehabilitation. The system used in this study offers a feasible solution to limitations of conventional models, particularly the lack of individualized task adjustment and limited capacity for monitoring patient engagement. By using real-time performance data to guide training, the approach reduces reliance on therapist supervision while preserving the benefits of personalized rehabilitation. This model may help expand access to cognitive therapy for patients who face mobility issues, transportation barriers, or live in regions with limited rehabilitation resources. Overall, the study provides clinically relevant evidence supporting the use of self-guided digital platforms as a complementary approach to traditional cognitive rehabilitation.

### 4.5 Limitations and future directions

This study has several limitations that should be considered when interpreting the findings. First, a subset of participants was unable to complete the TMT-A and TMT-B, and in accordance with standard scoring conventions, maximum scores were imputed for these cases. Although this approach is widely accepted, it may underestimate variability in cognitive performance among individuals with more severe impairments. Future studies may consider alternative analytic methods, such as censored data modeling or stratified subgroup analysis, to more accurately reflect such variations. Another limitation is that multiple secondary outcomes were analyzed without statistical correction for multiplicity. As such, these analyses should be interpreted as exploratory rather than confirmatory.

Second, the study employed a certain intervention period without long-term follow-up, limiting conclusions about the durability of treatment effects. Additionally, the sample size was modest and composed primarily of a homogenous population, which may restrict the generalizability of the results. Future research should include larger and more diverse samples, as well as longitudinal designs to assess sustained clinical effectiveness over time.

Third, because the intervention and control groups involved different participants and only the intervention group used the AI-driven system, it was not feasible to compare AI-generated task recommendations with therapist-selected tasks. This structural constraint precluded direct assessment of algorithm adherence. Future studies employing crossover or within-subject designs could allow for such comparisons. Moreover, the current system was based on rule-based logic rather than data-driven models; as more advanced AI technologies such as machine learning or deep learning become increasingly integrated into clinical practice, future iterations of adaptive rehabilitation systems may offer greater precision, flexibility, and therapist-level decision-making support.

Finally, although this study demonstrated the non-inferiority of AI-based telerehabilitation compared to therapist-supervised interventions through a direct comparison, further studies are warranted to examine long-term outcomes, cost-effectiveness, and subgroup-specific responses. These investigations will help refine the role of AI-driven rehabilitation in comprehensive stroke recovery strategies.

## Data Availability

The raw data supporting the conclusions of this article will be made available by the authors, without undue reservation.

## Footnotes

^1^ https://public.irb.or.kr/

^2^ https://cris.nih.go.kr/cris/index/index.do
